# Nightmare

**Published:** 1889-08-24

**Authors:** William Moore

**Affiliations:** K.C.I.E., late Surgeon-General with the Government of Bombay


### NIGHTMARE.

By Sir William Moore, K.C.I.E., late Surgeon-General with the Government of Bombay.

In a recent article on insomnia the advice of Confucius and Hippocrates was quoted, who both cautioned against sleeping on the back, in the posture of a dead man. Such a habit not only tends to sleeplessness, but it is also a fertile cause of bad dreams and of nightmare, especially when there is also derangement of the biliary or digestive organs. An old physician, Sir J. Floyer, said "incubus, or nightmare, is an inflation of the membranes of the stomach, which hinders the motion of the diaphragm, lungs, and pulse, with a sense of weight oppressing the breast." Burton calls it "incubus or witch-ridden," and the terminal word of nightmare is supposed to be derived from *mara*, a spirit, that in Northern mythology was presumed to torment or suffocate sleepers. The Greek term *ephialtes* conveys the idea of some living thing having taken its position on the breast, inspiring terror, impeding respiration, and paralysing the voluntary muscles. Many of the older authors have had something to say about incubus. Virgil, in the twelfth book of the Æneid, has given a description of it. "The vision said, and vanish'd from his sight, the dreamer wakening in a mortal fright," is a quotation from Dryden. Scott, in "The Lady of the Lake," had evidently nightmare in view, when he penned the lines:

> "In broken dreams the image rose,
> Of varied perils, pains, and woes.
> His steed now flounders in the brake,
> Now sinks his barge upon the lake.
> Now leader of a broken host
> His standards fall, his honour's lost.
> Then from my couch may heavenly light,
> Chase this worst phantom of the night."

It was probably nothing but attacks of incubus which gave rise to the complaint of Cowper, "To whatever cause it may be owing, whether to constitution or to God's express appointment, I am haunted by spiritual hounds in the night season." Another poet wrote: "In dreams they fearful precipices tread, or shipwrecked labour to some distant shore." It would thus almost seem that in former days nightmare was a more common complaint that it is now, for we do not find much mention of it in more modern books, either professional or otherwise. And that it was more common than it is now may be readily imagined, for we find it stated in an old medical work, "chesnuts and sour wine almost always produce incubus." Chesnuts and sour wine are not popular articles of diet among ourselves, or we should certainly hear more of nightmare than we now do.

Interruptions of sleep and nightmare may arise from such causes as asthma or heart affection, when the reason will probably be sufficiently evident. But as a general rule frightful dreams and incubus are the result in children of flatus, or "wind on the stomach," and in adults of derangements of the liver or biliary functions. No doubt an attack of nightmare may be excited by undigested food in the stomach, but when nightmare recurs there is nearly always some existing liver derangement, resulting probably in the elements of bile circulating in the blood and acting on the brain. Why dreams from such a cause should usually be of an unpleasant nature is no more evident than the actual and precise cause of dreams, which has yet to be explained. An author compared dreams with plays, as being a representation of something that does not really happen. Nevertheless, there are numerous authenticated instances of dreams "coming true." On this head Coleridge said: "Though most of the coincidences may be readily explained by the great and surprising power of imagination and association, yet it is impossible to say whether an inner sense does not really exist in the mind, seldom developed indeed, but which may have a power of presentiment." However this may be, when a person with nightmare is hanging by his toes from the corner of a high building, expecting to drop every moment, nothing so thoroughly satisfies him as the sudden discovery that he is safely at home in his bed. Dryden wrote, "glorious dreams stand ready to restore the pleasing shapes of all we saw before." But glorious or pleasant dreams are the perquisites of a healthy condition of system. Some persons hold the opinion that we always dream when asleep, and Locke said, "Those who sleep without dreaming can never be convinced that their thoughts were busy without their knowing it." Probably pleasant dreams are not always remembered, or only partially so. But when we suffer from incubus there is generally a vivid recollection of it, whether in the form of a black dog, as Forestus of old experienced or in the shape of falling from a balloon, as latterly related by a person who had recently seen a "professor" descend in his parachute. It is reported that the savages of Mount Atlas were dreamless; but if so, they are the only savages who could thus boast. In our experience, savage and semi-civilised men dream even more than the products of civilisation. And the reason is evident. They fill their stomachs to repletion and, like animals, sleep immediately afterwards. Whatever ills savages escape, such as taxes, chimney-pot hats, barrel organs, and trade circulars, they do not escape incubus; and as of old, it was attributed to spirits (*succubi* in women, *incubi* in men), so now among savage races it is regarded as the work of demons in possession, instead of being attributed to the true cause, repletion.

Nervous and hypochondriacal temperaments, indigestible food, sedentary employment, too much mental work, acidity and flatus, pregnancy, lying on the back, will all excite occasional nightmare. But also, most of the causes tend to stomach and liver derangements. An alkali, as carbonate of soda, or Burrough's and Wellcome's compressed soda-mint tabloids, are good temporary remedies. Habitual frightful dreams, or nightmare, however, demands more energetic treatment. As a general rule, medicines which are known to act on the liver, and to excite a flow of bile, are necessary, combined with care as regards diet, temperance, both as regards food and drink, plenty of fresh air and exercise in the daytime, and adherence to the old adage, "Early to bed and early to rise."

